# Investigation of Content, Stoichiometry and Transfer of miRNA from Human Neural Stem Cell Line Derived Exosomes

**DOI:** 10.1371/journal.pone.0146353

**Published:** 2016-01-11

**Authors:** Lara Stevanato, Lavaniya Thanabalasundaram, Nickolai Vysokov, John D. Sinden

**Affiliations:** 1 Stem Cell Discovery, ReNeuron, Guildford, United Kingdom; 2 Wolfson CARD, Kings College London, Guys Campus, London, United Kingdom; University of Bristol, UNITED KINGDOM

## Abstract

Exosomes are small (30–100 nm) membrane vesicles secreted by a variety of cell types and only recently have emerged as a new avenue for cell-to-cell communication. They are natural shuttles of RNA and protein cargo, making them attractive as potential therapeutic delivery vehicles. MicroRNAs (miRNAs) are short non-coding RNAs which regulate biological processes and can be found in exosomes. Here we characterized the miRNA contents of exosomes derived from human neural stem cells (hNSCs). Our investigated hNSC line is a clonal, conditionally immortalized cell line, compliant with good manufacturing practice (GMP), and in clinical trials for stroke and critical limb ischemia in the UK (clinicaltrials.gov: NCT01151124, NCT02117635, and NCT01916369). By using next generation sequencing (NGS) technology we identified the presence of a variety of miRNAs in both exosomal and cellular preparations. Many of these miRNAs were enriched in exosomes indicating that cells specifically sort them for extracellular release. Although exosomes have been proven to contain miRNAs, the copy number quantification per exosome of a given miRNA remains unclear. Herein we quantified by real-time PCR a highly shuttled exosomal miRNA subtype (hsa-miR-1246) in order to assess its stoichiometry per exosome. Furthermore, we utilized an *in vitro* system to confirm its functional transfer by measuring the reduction in luciferase expression using a 3’ untranslated region dual luciferase reporter assay. In summary, NGS analysis allowed the identification of a unique set of hNSC derived exosomal miRNAs. Stoichiometry and functional transfer analysis of one of the most abundant identified miRNA, hsa-miR-1246, were measured to support biological relevance of exosomal miRNA delivery.

## Introduction

Exosomes are small membrane vesicles that originate from multi-vesicular bodies and are secreted by a variety of cell types [1, 2]. Recent findings indicated that the transfer via exosomes of genetic information, such as mRNAs and microRNAs (miRNAs) [3, 4], can modulate cellular activities in recipient cells. MiRNAs are a class of non-coding RNA (ncRNA) species of about 22 nucleotides in length that functionally repress target mRNA by binding to their 3’ untranslated regions (3’UTR). MiRNAs are involved in several biological processes, such as proliferation, development, differentiation and apoptosis; a single miRNA can potentially target hundreds of genes [5–7]. MiRNA transfer via exosomes could bypass recipient cell transcriptional controls providing a relatively direct means of regulation. Studies, delivering purified exosomes to recipient cells, have reported transfer of miRNAs in experimental settings and support their use as a potential therapeutic strategy [8, 9]. Although the possibility of exosome-mediated miRNA transfer as a mode of intercellular communication is an attractive concept, the biological significance of miRNA transfer to target cells needs to be clarified [10]. Quantification of key components is fundamental to testing the validity of any model [11]. Stoichiometric analysis of exosomal miRNA copy number contents might provide insight into the significance of miRNA-based intercellular communication.

As exosomes are sub-diffraction limit particles, their quantification is challenging to address, and therefore cannot be directly enumerated by light microscopy or flow-cytometric methods [12]. Although electron microscopy is typically utilized to visualize exosomes [2, 13], its quantitative utility is limited by the variation of complex sample preparation processes. Recently, nanoparticle tracking analysis (NTA) has been utilized as an innovative system for quantification and sizing of particles from about 30 to 1,000 nm [14] by visualizing the particle light scattering and using it to track their Brownian motion for estimation of the size distribution through the Stokes-Einstein equation [12].

Technological advances have generated a multitude of platforms for the systematic evaluation of miRNAs. These new tools are largely based on mRNA expression analysis and array-based comparative genomic hybridization. Recently, next generation sequencing (NGS) technology is challenging microarrays as the tool of choice for analysis of genomics [15, 16]. Rapid advancement of this technology has made it possible to study expression profiles of miRNAs comprehensively and efficiently. Furthermore, NGS allows for the simultaneous discovery of new miRNAs and confirmation of known miRNAs together with the evaluation of differential expression between samples [17, 18].

The understanding of the relationship between the miRNA and the seed region has enabled the development of several bioinformatics tools to scan mRNA 3’UTRs and predict putative target genes. Once candidate miRNA/target gene pairs are identified their functional analysis can be confirmed experimentally by using miRNA mimics. MiRNA/target gene 3'UTR mediated down-regulation can be measured using a dual luciferase reporter *in vitro* assay [19].

In this study we set out to determine the content of extracellular miRNAs that are associated with exosomes purified from conditioned media (CM) of a clinical grade human neural stem cell (hNSC) line, designated CTX0E03 [20–22]. By utilizing NGS we identified a set of exosomal miRNAs that are enriched compared to cellular ones. By adopting quantitative methods we directly determined the stoichiometry of a selected highly enriched miRNA. By developing an *in vitro* assay we assessed exosomal miRNA functional transfer. Thus it can be concluded that exosomes might mediate miRNA delivery and could be used as therapeutics in their own right.

## Materials and Methods

### Cell line and culture reagents

CTX0E03/hNSC is of human origin and established as a clonal cell line by conditional immortalization with c-mycER^TAM^, and clonal selection as described in Pollock *et al*. [22]. HNSCs were cultured in a serum-free medium (RMM) [22] supplemented with epidermal growth factor (EGF, 20 ng/ml, Peprotech), basic fibroblast growth factor (bFGF, 10ng/ml, Peprotech) and 4-hydroxytamoxifen (4-OHT, 10 mM, Sigma) on laminin (20 μg/ml, or 2.28 μg/cm^2^ AMS Biotech) coated vessels and incubated at 37°C in a humidified atmosphere containing 5% CO_2_ [22].

### Isolation of exosomes

Exosomes were isolated from hNSC culture supernatants by using a protocol modified from Thery et al [2]. Conditioned media (CM) were collected from 80–90% confluent hNSC in sterile conditions. CMs were filtered using a filter unit (Millipore, SCGPU05RE) with a 0.22 μm membrane to remove intact cells and cell debris. Ultracentrifugation was performed at 120,000 g (Sorvall WX ULTRA SERIES, rotor A-641) for 2 hours at 4°C. Pellets were resuspended and washed in 1 ml of cold PBS and ultracentrifuged again (120,000 g, 2 h, 4°C). The pellets were finally resuspended in 100 μL of cold PBS, transferred into a low binding protein tube and immediately stored at -80°C.

### Nanoparticle tracking analysis (NTA)

NTA measurements were performed using a NanoSight LM10 instrument (NanoSight NTA 2.3 Nanoparticle Tracking and Analysis Release Version D) following the manufacturer's instructions. The NanoSight LM10 instrument measures the rate of Brownian motion of nanoparticles and consists of a light scatter detection system that provides a reproducible platform for specific and general nanoparticle characterization. NTA acquisition settings were optimized and kept constant between sample readings.

### QNano particles analysis

Nanoparticle size distribution and particle quantification were performed by qNano system (Izon, Christchurch, New Zealand) in accordance with the manufacturer’s instructions. The qNano technology is based on the Coulter Counter Principle. A polyurethane-based nanopore is immersed in electrolyte and a voltage is applied to create an electrical field and a particle flow. When the particles pass through the pore one by one, they create a temporary drop in current, a ‘blockade event’, which is dependent on their volume. Blockade events were calibrated against particles of a known size measured under identical settings.

### Western blotting (CD63, CD81, MYC, and FAM53C)

Proteins from exosomes and hNSCs were separated by SDS polyacrylamide gel electrophoresis on NuPage^®^ 10% Bis-Tris polyacrylamide gels following manufacturer’s guidelines (Life Technologies), then transferred to Invitrolon^™^ PVDF membranes (Life Technologies), blocked using WesternBreeze blocker/diluent (Life Technologies) and probed with primary mouse monoclonal anti-CD63 and anti-CD81 (2 μg/ml, Life Technologies), previously described exosome enriched markers [23], and anti-c-MYC monoclonal antibody (1:100 dilution, Santa Cruz Biotechnology Inc), a hNSC marker. Proteins from untreated and scrambled miRNA (AllStars negative control siRNA AF 488, Qiagen), hsa-miR-1246 mimic (Qiagen), and exosome treated HeLa were separated and blotted as described above, and probed with primary rabbit polyclonal anti-FAM53C (1:100, Sigma) and primary mouse monoclonal α-tubulin (1:1000, Sigma). Proteins were detected using anti mouse (1:1000, Pierce Biotechnology Inc.) or anti–rabbit (1:1000, Cell Signaling Technology) horseradish peroxidase-conjugated secondary antibody accordingly and developed with enhanced chemiluminescence reagent (Thermo Scientific). Western blot images were captured using BioRad FluorS Imaging Unit and densitometry carried out using ImageJ software (National Institutes of Health).

### Next generation sequencing

NGS analysis was performed by GATC Biotech (Germany) and required the preparation of a tagged miRNA library for each sample followed by sequencing and miRBase scanning.

#### Preparation of cellular and exosomal total RNA

Total RNA was purified using miRNeasy (Qiagen) according to manufacturer’s instructions. The concentration of total RNA was determined using a NanoDrop spectrophotometer (Thermo Scientific) and total RNA was analyzed using 2100 Bioanalyzer (Agilent).

#### Construction of tagged miRNA libraries

Sequencing libraries were generated by ligation of specific RNA adapter to both 3’ and 5’ ends for each sample followed by reverse transcription, amplification, and purification of small-RNA libraries (size range of contained small-RNA fraction 22–30, nt).

#### Sequencing on an Illumina HiSeq 2000 (single read)

Analysis of one pool includes up to 45,000,000 single reads, and each read length is up to 50 bases. Sequencing was quality controlled by using FastQ Files (sequences and quality scores).

#### Identification of known miRNAs was performed as followed

RNA adapters were trimmed from resulting sequences and raw data cleaned. Raw data were clustered and for each cluster the number of reads was provided. MiRNAs were identified by miRBase scanning. The NGS was performed in two batch preparations of hNSCs and exosomes.

### Quantitative real-time PCR (qRT-PCR)

Total RNA was isolated from all samples using the miRNeasy Kit (Qiagen) according to the manufacturer’s protocol. A defined amount of cel-miR-39-3p *Caenorhabditis elegans* oligo-ribonucleotide was added at the same time as the Qiazol reagent to assess efficiency of total RNA recovery. CDNA synthesis was performed using SuperScript^™^ II RT (Invitrogen) according to manufacturer’s instructions. QRT-PCR was performed using the lightcycler LC480 system (Roche Diagnostics), specific primer set (Forward- hsa-miR-1246: aatggatttttggagcagg, and cel-miR-39-3p: tcaccgggtgtaaatcagcttg, and Reverse- Universal primer, Qiagen) and miScript PCR Starter kit (Qiagen) according to manufacturer’s instructions. The following conditions, 95°C for 5 s, followed by 35 cycles at 95°C for 10 s, 60°C for 20 s, 72°C for 20 s (measuring the fluorescence) were used for the qRT-PCR. At least three biological replicates were used. In order to carry out absolute miRNA quantification, a serial dilution of known concentration of miRNA mimic was used to plot a standard curve.

### Transfections and dual luciferase assay

Transient transfections of HeLa cells were performed using Lipofectamine 2000 (Invitrogen) according to the manufacturer’s instructions. For the validation of a miR-1246 putative target, FAM53C 3’UTR, HeLa cells were plated at a density of 10^4^ cells/well in 96-well plates and co-transfected with 100 ng of MiTarget^™^ MicroRNA 3'UTR Target Clone HmiT059263-MT01 plasmid (GeneCopoeia, FAM53C/ NM_001135647.1 3’UTR) and a range of dilution of miR-1246 mimic. Control wells were co-transfected with HmiT059263-MT01 plasmid and AllStars negative control siRNA AF 488 (Qiagen). The efficiency of miRNA mimic and plasmid transfections were found to be close to 100% [19]. For exosomal functional miRNA transfer assessment HeLa cells were pre-treated with exosomal preparations followed by transfection with FAM53C 3’UTR dual luciferase plasmid. Control wells were transfected with the FAM53C 3’UTR dual luciferase plasmid and either positive (miR-1246 mimic, 120nM) or negative (AllStars negative control siRNA AF 488, scrambled miRNA, 120nM) miRNAs. HmiT059263-MT01 plasmid expresses both *firefly* and *renilla* luciferase [19]. *Firefly* and *renilla* luciferase activities were measured 24 hrs after transfection using the Luc-Pair miR luciferase assay (GeneCopoeia) and a GloMax^™^ 96 Microplate Luminometer (Promega). *Firefly* luciferase activity was normalized to *Renilla* activity for each transfected well and expressed as percent of control transfections.

For the evaluation of FAM53C protein expression by Western blotting, HeLa cells were transiently transfected with hsa-miR-1246 mimic and AllStars negative control siRNA AF 488 as described above or treated with hNSC exosomal preparation; untreated HeLa cells were use as control.

At least three biological replicates were analyzed.

### Statistical analysis

Where applicable, the data were analysed by Student's t-test and p<0.05 was considered statistically significant. All error bars indicate ± standard error of the mean (SEM).

## Results

### Exosome isolation and characterization

**Fig 1:**
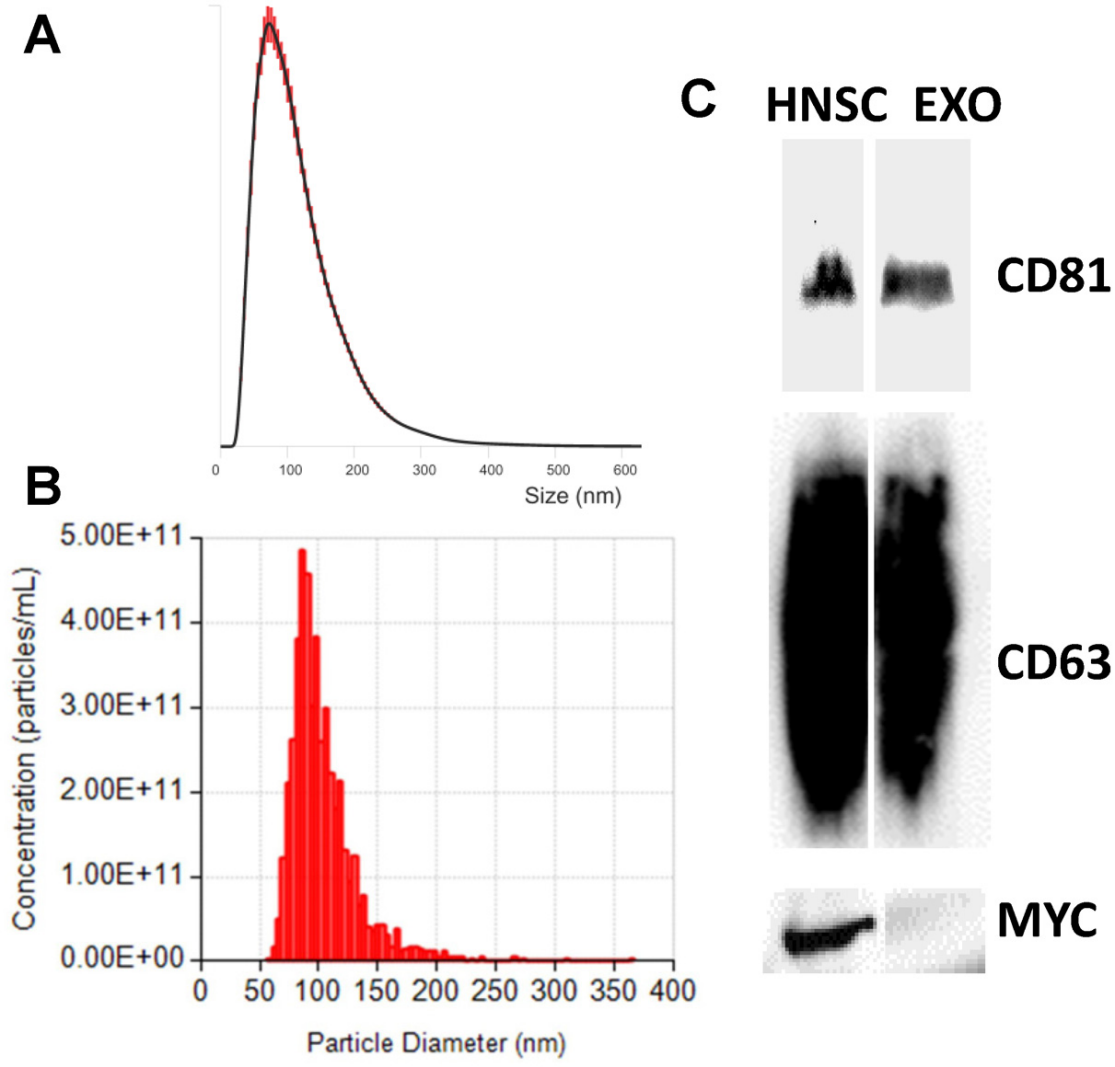
The characterization of exosomes derived from hNSCs. Size distribution of exosomes analyzed with the NTA (A) and qNano (B), representative traces. In agreement with exosome sizes, isolated exosomes had a mode of approximately 100 nm. (C) Molecular characterization of exosomes and producer hNSCs by Western blotting. Protein extracts from hNSCs and exosomes were assessed using antibodies against exosomal protein markers (CD81 and CD61), and hNSC protein marker (MYC).

Exosomes were purified by an ultracentrifugation protocol [2] from CM of the clinical grade clonal hNSC line, CTX0E03 [22]. To ensure that we isolated exosomes we conducted Western blotting on our preparations and confirmed the presence of well-established exosomal markers, CD63 and CD81 tetraspanins [23], and the absence of a cellular marker, MYC; CTX0E03 hNSCs were conditionally immortalized with c-mycER^TAM^ [22] (Fig 1C). These findings are consistent with purified exosome samples. Additionally, we further analyzed size distribution and number of particles per μl of exosome preparations using NTA and qNano. Based on these measurements and the shoulder associated with the major distribution peak (Fig 1A and 1B), our preparations have a modal size of 95 nm ± 2.3 SEM (NTA) and 86 nm ± 2.2 SEM (qNano) and confirmed populations with particle sizes consistent with exosomes.

### Next generation sequencing for miRNA

**Fig 2:**
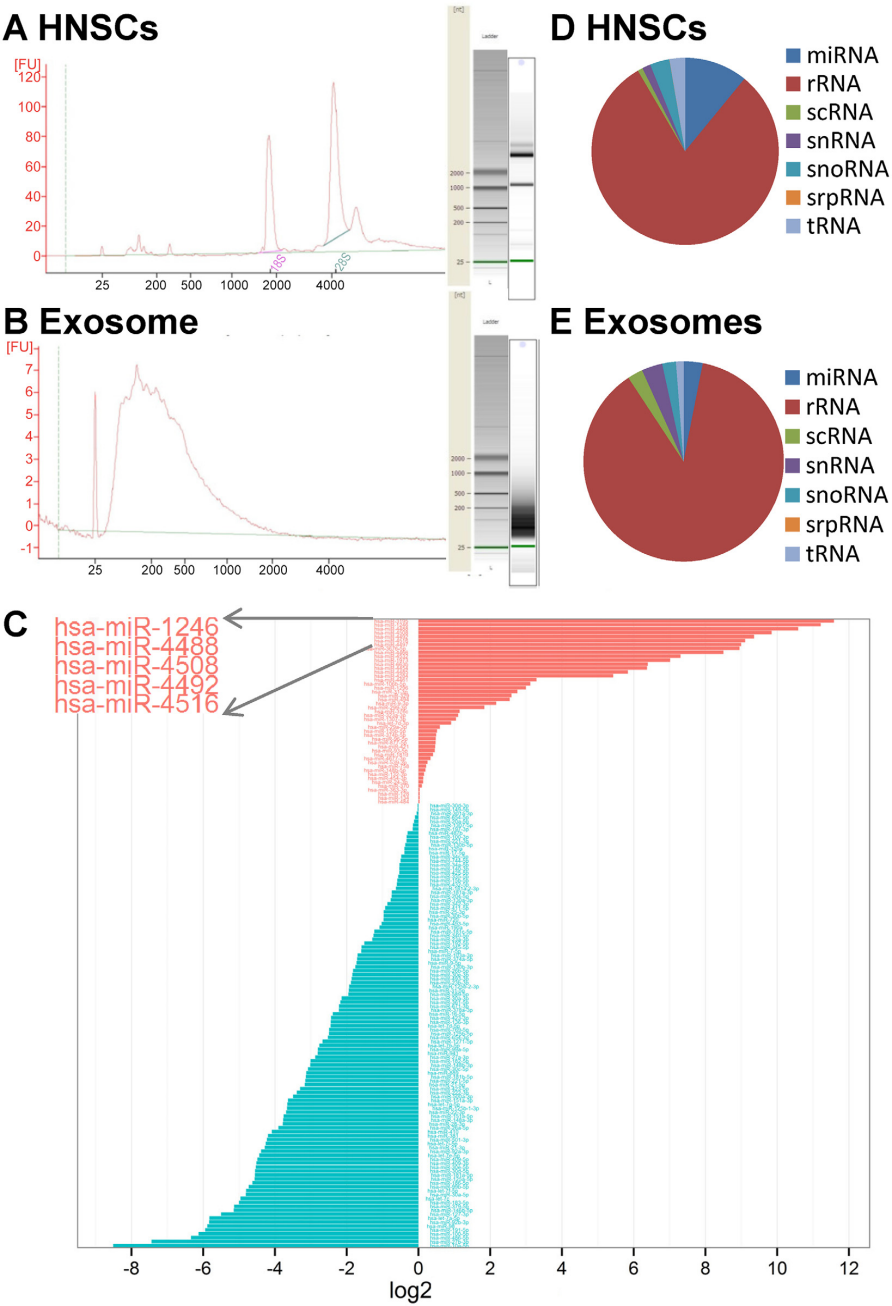
MiRNA next generation sequencing. Cellular (A) and exosomal (B) total RNAs were processed by an Agilent 2100 Bioanalyzer. The corresponding virtual gel images generated by the software are depicted as electropherograms. (C) Representative diagram of differential miRNA distribution in exosomes compared to hNSC producers. MiRNA types preferentially released in exosomes are presented in red or retained within the hNSCs in blue, data expressed as log_2_ ratio of exosomal/cellular miRNAs normalized read counts. Pie chart representation of the distribution of small RNA categories in hNSC (D) and exosome (E) samples.

Total RNA extractions were performed using miRNeasy kit and analyzed using a Bioanalyser (Fig 2A and 2B). The RNAs sized between 15 and 70 nucleotides (nt) were used to construct small RNA cDNA libraries. The libraries were read with the Illumina platform. Sequences were mapped based on their overlaps with publicly available human genome (HG19) and miRBase V19 [24]. For each miRNA sequence-based profile, the number of sequence reads was used to estimate expression level of each miRNA. Differential miRNA uptake in the exosomes compared to cell producers is represented in Fig 2C and S1 Table. Hsa-miR-1246, hsa-miR-4488, hsa-miR-4508, hsa-miR-4492 and hsa-miR-4516 were identified as the 5 most abundant miRNA types in exosomes derived from hNSC producers and had the highest ratio of exosomal to cellular miRNA abundance. According to this finding and as previously reported [15], dissemination of miRNAs into the exosomes does not appear to be a random process, but that cells actively sort selective miRNAs for extracellular destination. In the exosomes 113 known miRNAs were identified compared to 446 in hNSCs.

The reads obtained were mapped against known small RNA libraries to identify sequences originating from sources such as rRNA, tRNA, snRNA and snoRNA. Overall in the exosomes 3.10% was found to map miRNA, 3.35% snRNA, 2.47% scRNA, 2.21% snoRNA, 87.65% rRNA, 0.02%, srpRNA, and 1.20% tRNA and in the hNSC, 29.52% was found to map miRNA, 2.25% snRNA, 0.95% scRNA, 0.97% snoRNA, 61.55% rRNA, 0.02%, srpRNA, and 4.74% tRNA, respectively (Fig 2D and 2E). Comprehensively, a significant portion of the sequencing reads was from rRNA and tRNA species [25]. NGS files can be found in S1 Table.

### Quantification of copy of miRNA per exosomes

**Fig 3:**
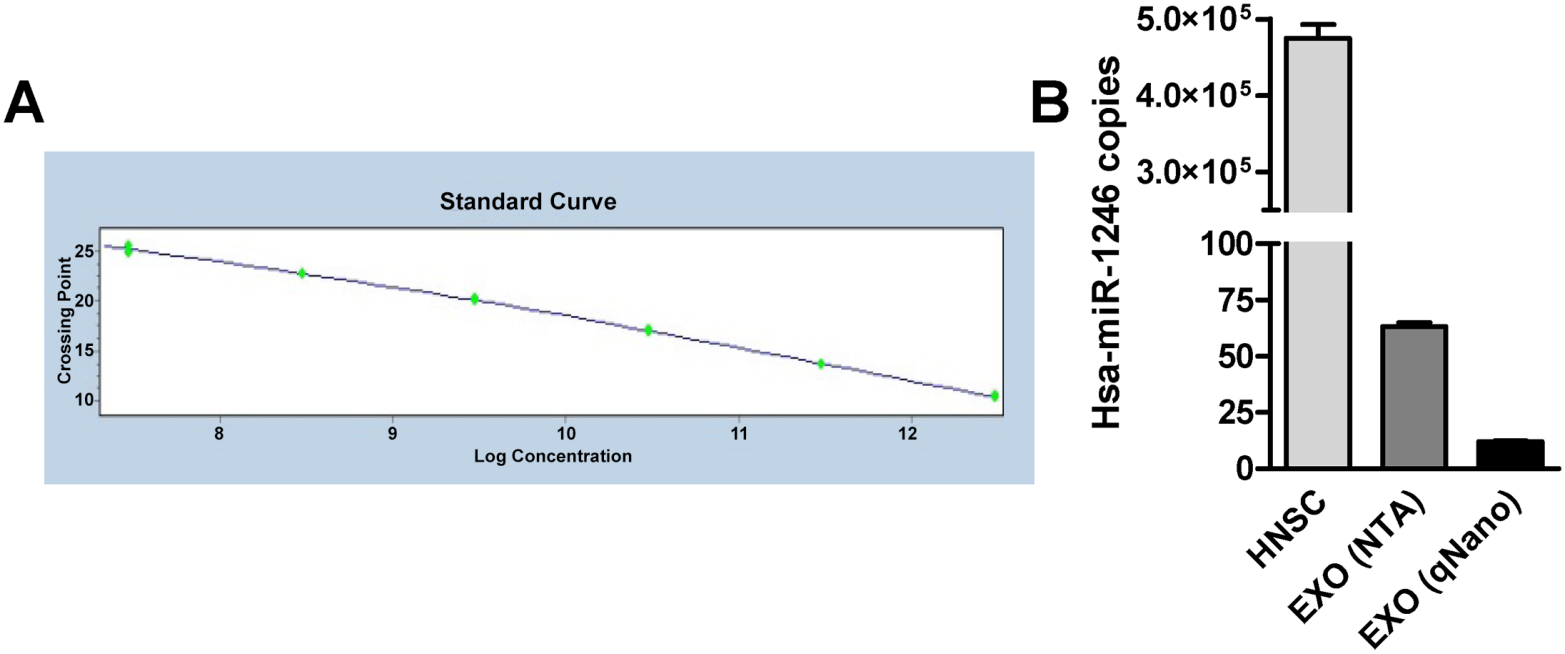
Absolute quantification of selected miRNA. (A) Example of standard curve obtained for miRNA quantification. MiRNA mimic was diluted to a concentration range of 3.01×10^12^–3.01×10^7^ copies per well. Each point plotted is an average of triplicate fluorescence values for each standard concentration measured. (B) Diagram showing the quantification of hsa-miR-1246 copy number per hNSC and per exosome (EXO). Exosome particle quantification was performed using two independent methods, NTA and qNano; cell number quantification was performed by hemocytometer analysis. The error bars represent ± SEM.

The qRT-PCR absolute quantitation system requires that the absolute quantities of the standards are first known by some independent means. Known concentrations of investigated miRNAs, measured by nucleic acid A260 nm spectrophotometry, were converted to copy numbers using their miRNA molecular weights and used to produce a standard curve. The absolute concentration of the investigated miRNA in any tested samples was determined by the simple interpolation of its PCR signal (crossing point, Cp) into this standard curve by using LightCycler^®^ 480 Software 1.5 (Roche) at completion of qRT-PCR run. One of the most enriched miRNA, hsa-miR-1246, was selected and its copy number per exosome was quantified by qRT-PCR absolute quantification (Fig 3A). Furthermore, a synthetic non-human cel-miR-39 was spiked in during RNA isolation. QRT-PCR absolute quantification of cel-miR-39 copy numbers were quantified to assess that the efficiency of cel-miR-39 recovery was similar among tested samples and consequentially the quantification of hsa-miR-1246 was not biased during the experimental procedures. The number of exosomal particles per preparation was measured either using NTA or qNano. We opted for two independent quantifications to overcome a lack of standards for optimal quantification of exosomal particles. Approximately a 5-fold difference in particle concentration was found using NTA and qNano. Overall, at least 10 copies of miRNA were found per exosome (63.21 ± 1.64 SEM, NTA, and 12.08, ± 0.32 SEM, qNano) compared to 4.75×10^5^ ± 18×10^3^ SEM copies per hNSC (Fig 3B).

### *In vitro* functional miRNA transfer assay

**Fig 4:**
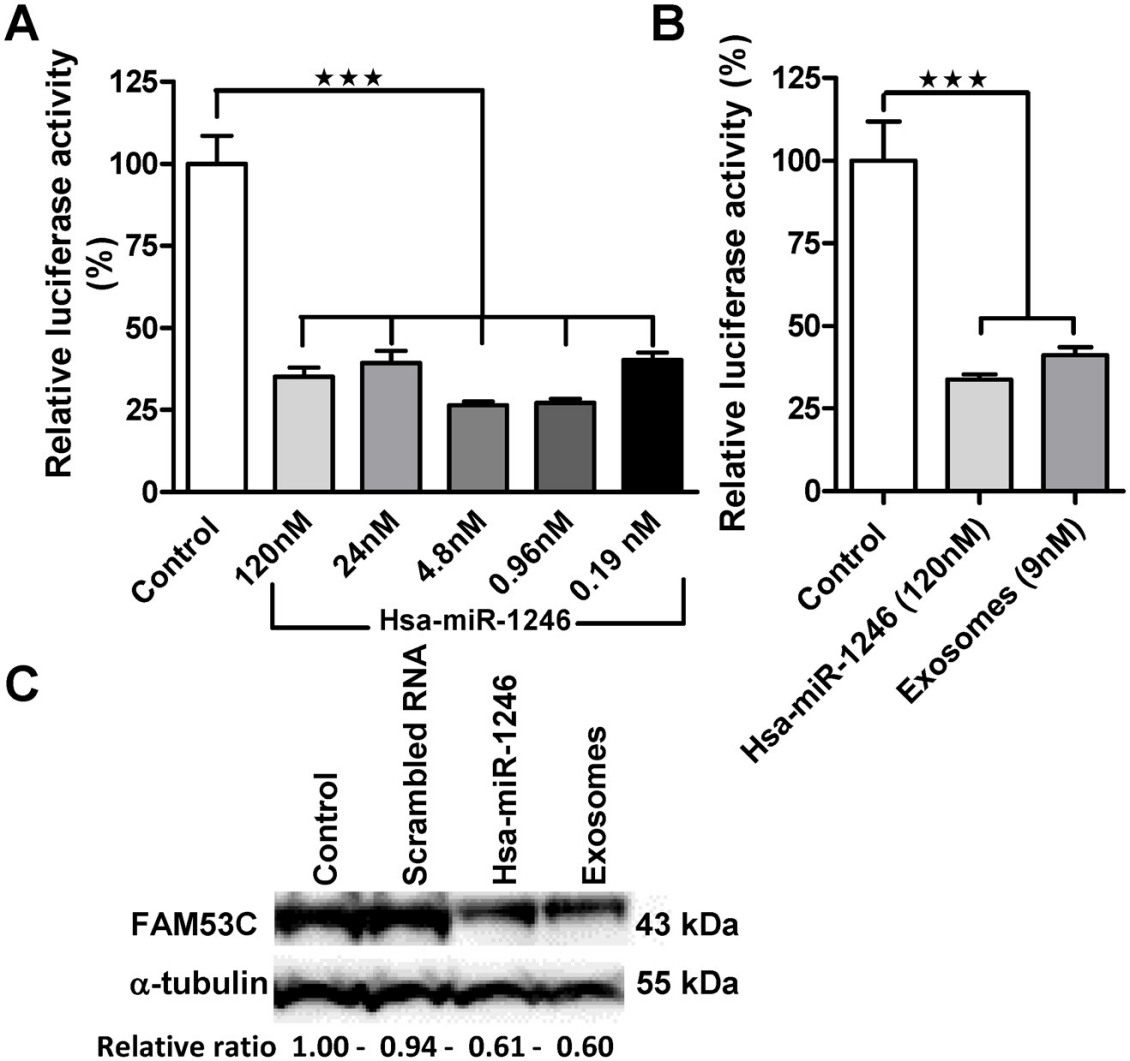
Functional transfer assessment of exosomal miRNA. (A) *In vitro* assay validation of miR-1246/ 3’UTR-FAM53C mRNA binding was evaluated by measuring the relative luciferase reduction activity caused by the co-transfection of HeLa cells with the dual luciferase reporter plasmid and a range of dilution of hsa-miR-1246. Data were expressed as percent of control (scrambled miRNA) transfections, n = 6. (B) Biological functional transfer assessment of exosomal hsa-miR-1246 cargo. HeLa cells were pre-treated with purified exosomes and transfected with FAM53C 3’UTR dual luciferase plasmid. Relative reduction in luciferase activity in hsa-miR-1246 mimic and exosome treated samples was expressed as percent of control (scrambled miRNA) transfections, n = 6. The error bars represent ± SEM; *** p<0.001. (C) Western blot analysis of FAM53C in untreated (control), scrambled miRNA, hsa-miR-1246 mimic and exosome treated HeLa. FAM53C expression levels were normalised to α-tubulin following quantification by densitometry (ImageJ) and presented as relative ratio to the control sample.

A number of computational algorithms have been developed to predict miRNA target mRNA. Some of the more well-known prediction algorithms include PicTar (http://pictar.mdc-berlin.de/) [7], TargetScan (www.targetscan.org) [26], and miRanda (www.microrna.org) [6]. A single miRNA can recognize hundreds of targets; FAM53C was identified as the top ranking target mRNA of hsa-miR-1246 by a number of prediction algorithms. HeLa cells and dual luciferase (*Firefly* and *Renilla*) reporter plasmid were selected for the development of a functional miRNA transfer analysis assay. Dual luciferase relies on two different reporter genes in the same plasmid and offers an improved system allowing normalization for effects caused by suboptimal transfection and cytotoxic effects compared with a single luciferase plasmid system. As part of our assay development we assessed hsa-miR-1246/ FAM53C target validation by co-transfecting HeLa cells with a range of dilution of hsa-miR-1246 mimic and FAM53C 3’UTR dual luciferase plasmid. We observed a reduction in relative *firefly* luciferase activities ranging from 73.59% ± 1.15 SEM to 59.77% ± 2.32 SEM compared to the negative miRNA control (Fig 4A). For exosomal functional miRNA transfer assessment HeLa cells were pre-treated with exosomal preparations followed by transfection with FAM53C 3’UTR dual luciferase plasmid. Control wells were transfected with FAM53C 3’UTR dual luciferase plasmid and either positive or negative miRNAs. We detected a 58.79% ± 2.39 SEM significant reduction (p < 0.005) in relative luciferase activity (Fig 4B) compared with the negative miRNA control. In the exosome treated samples, based on its absolute quantification, hsa-miR-1246 final concentration per treated well was calculated to be 9 nM and within the assay development dose range (120–0.19 nM). Furthermore we performed Western blot analysis of FAM53C. The differential expression of this protein was verified in hsa-miR-1246 mimic and exosome treated HeLa cells compared with untreated and scrambled miRNA treated HeLa cells (Fig 4C).

## Discussion

Multiple reports have implicated exosomes as a vehicle for cell–to-cell communication through carriage and transfer of miRNAs between cells [3, 4, 27]. This discovery has generated a great interest in these vesicles as potential vehicles for regenerative and therapeutic miRNAs [28, 29]. However, the identification of miRNA contents has a tendency to be biased by the choice of detection system analysis. Herein, we opted for the NGS technology. NGS is now challenging microarrays as the tool of choice for genomics analysis because it is not biased by the requirement of prior knowledge of the genetic query [30].

We first investigated the mRNA signatures of hNSCs and their exosomes by small RNA sequencing analysis (NGS). We found a number of differentially expressed miRNAs in hNSCs compared with hNSCs-derived exosomes and generated a differential expression profile. Hsa-miR-1246, hsa-miR-4488, hsa-miR-4508, hsa-miR-4492 and hsa-miR-4516 were identified as the 5 most exosomal enriched miRNAs (Fig 2C). Interestingly, these miRNAs were not previously reported when using microarray analysis in a similar stem cell type [31]. This finding supports the use of NGS as a suitable technique for unbiased identification of exosomal miRNA content.

The highly enriched exosomal miRNAs may have significant therapeutic impacts on target cells. Hsa-miR-1246, one of the most abundant exosomal miRNAs in our hNSC, was originally identified following miRNA sequencing in embryonic stem cells [32]. In recent years hsa-miR-1246 has been described as a novel p53 target miRNA [33]. Hsa-miRi-1246 expression has been associated as a pro- [34] and anti-oncogenic [35] biomarker and related to Down's syndrome [36]. Overall miR-1246 has been demonstrated to play an essential role in regulating cell growth and apoptosis [37, 38].

However, stoichiometric analyses of exosome-mediated miRNA communication have been under-addressed in the past. In a previous study [10], even the most abundant miRNAs were reported to be far less than one copy per exosome, suggesting a lack of exosomal potential as mediator of a therapeutic miRNA transfer. On the other hand the RNAi therapeutics world is shedding some light on dose-response requirements for the clinical relevance of miRNAs or small interfering RNAs (siRNAs); for example a doses of approximatively 100 nM are used for *in vitro* proof of concept studies [39] and in the range of mg/kg for clinical applications [40, 41]. Bearing in mind these observations, we targeted our investigation to quantify the miRNA stoichiometry of a selected miRNA and verify that hNSC exosomal preparations could transfer a certain miRNA at a functional level sufficient to mediate a biological effect. For this we developed a hsa-miR-1246/ FAM53C *in vitro* reporter luciferase assay in HeLa cells in order to measure the reduction of luciferase protein expression driven by the 3’UTR FAM53C [15] following exposure to hNSC exosomal preparations and hsa-miR-1246 mimic.

Interestingly, based on the qRT-PCR absolute quantification of hsa-miR-1246 copy number per cell (4.75×10^5^) and the ratio of the exosome and cell volumes (2.33x10^-04^ μm^3^/380 μm^3^), we would have expected approximately 0.5 copies per exosome. This finding is consistent with a previous report (9). However, NGS analysis of miRNA contents exhibited a preferential enrichment of a subset of miRNAs in the exosomes and this supports our stoichiometry finding of at least 10 copies of a selected miRNA per exosome (Fig 3B). It is important to emphasize that by using different quantification tools (NTA and qNano) we observed a discrepancy in the particle quantification. The International Society for Extracellular Vesicles (ISEV) is addressing the requirement for standardization of quantification in the exosome field [42]. As a validation of presumptive functional transfer of our stoichiometric finding, we developed an *in vitro* method to confirm empirically that the exosome preparation could transfer a functional moiety of miRNA. By using the 3’UTR luciferase reporter gene assay [43] we confirmed that miRNA level is physiologically relevant and showed that exosomal miRNAs can access the molecular machinery of miRNA-mediated gene repression upon transfer into recipient cells (Fig 4B).

In summary, attempts have recently been made to characterize exosomal miRNA content and functionality for therapeutic applications [28]. However, there is limited collective information on miRNA subtypes and their stoichiometry per exosome. Here we have reported exosomal miRNA subtype identification, a quantitative evaluation of a selected exosomal miRNA stoichiometry, and its transfer. These data could provide valuable insight into the mechanism and physiologic relevance of hNSC derived exosomal miRNAs and their suitability for therapeutic applications.

## Supporting Information

S1 Table Differential miRNA uptake in the exosomes compared with hNSC producers. The differential miRNA uptake was calculated as Log2 fold change of the ratio (RPM exosome samples / RPM cell producer samples); RPM stands for reads per million.
(XLSX)
